# The mediating role of career concerns between career goal inconsistency, alienation and loneliness

**DOI:** 10.3389/fpsyg.2025.1670414

**Published:** 2025-09-24

**Authors:** Cemal Özman, Sinan Uğraş, Ahmet Enes Sağın, Mehmet Akif Yücekaya, Ender Eyuboğlu, Sonay Serpil Daşkesen

**Affiliations:** ^1^Department of Sport Management, Faculty of Sport Sciences, Bartın University, Bartın, Türkiye; ^2^Department of Physical Education and Sports Teaching, Faculty of Sport Sciences, Çanakkale Onsekiz Mart University, Çanakkale, Türkiye; ^3^Coaching Education Department, Faculty of Sport Sciences, Bartın University, Bartın, Türkiye; ^4^Department of Sports Management, School of Physical Education and Sports, Dicle University, Diyarbakır, Türkiye; ^5^Department of Physical Education and Sports Teaching, Faculty of Sport Sciences, Atatürk University, Erzurum, Türkiye

**Keywords:** career goal inconsistency, career concerns, loneliness, alienation from university, sports sciences

## Abstract

Uncertainty and inconsistency in career goals can weaken students’ psychosocial adjustment. While the existing literature reports a link between this situation and loneliness and alienation from university, evidence on how these connections develop, particularly among sports science students, is limited. This study examined the mediating role of career concerns in the relationship between career goal inconsistency, loneliness, and university alienation. The study included 631 students from the sports science faculty in Türkiye. Validated self-report scales were used to measure the variables. The mediation analysis was carried out through the JASP 0.16.4 statistical program using structural equation modelling and bootstrap procedures. The tested model was confirmed. Career goal inconsistency positively associated with career concerns (*β* = 0.46, *p* < 0.001), loneliness (*β* = 0.21, *p* < 0.001), and alienation from university (*β* = 0.26, *p* < 0.001). Career concerns also associated with loneliness (*β* = 0.17, *p* < 0.001) and alienation (*β* = 0.15, *p* < 0.001). Indirect associations were significant for the paths from career goal inconsistency to loneliness (*β* = 0.08, *p* < 0.001) and to alienation (*β* = 0.07, *p* = 0.001) through career concerns. The explained variances were *R*^2^ = 0.36 for career concerns, *R*^2^ = 0.16 for loneliness, and *R*^2^ = 0.20 for alienation. These findings suggest that inconsistencies in career goals are associated with increased students’ career anxiety, loneliness, and alienation from university, both directly and indirectly. Therefore, it is important to provide career counselling and guidance services that reduce students’ anxiety and strengthen their sense of belonging to the university, especially in fields such as sports sciences, where career paths are often uncertain.

## Introduction

Career planning is a multifaceted process that influences individuals’ ongoing professional growth and presents significant challenges, including identity formation, goal setting, and the cultivation of professional self-confidence, particularly for university students. The uncertainties and obstacles encountered throughout this process can significantly impact a person’s social and psychological well-being. When there is a misalignment between an individual’s current academic or occupational position and their long-term career goals, this is referred to as career goal inconsistency. This condition has been associated with adverse mental outcomes such as loneliness, alienation, and anxiety (Diaconu-Gherasim et al., 2024; Haratsis et al., 2015; Hardie, 2014; Hyvönen et al., 2011). Research indicates that such inconsistencies may undermine social interactions, reduce the sense of belonging, and be associated with affective states like loneliness (Walton and Cohen, 2007; Ozcelik and Barsade, 2018). Sense of belonging refers to the perception of being accepted, valued, and included within a social or academic environment. It is a key factor in individuals’ motivation and psychological adjustment (Allen et al., 2021).

Alienation is characterised by an individual’s disconnection from their academic and social surroundings, typically resulting in diminished motivation, social isolation, and poor academic performance (Jiang et al., 2024). A weakened sense of belonging is positively associated with heightened feelings of alienation, often leading to socio-emotional withdrawal (Morinaj and Hascher, 2019). Limited social interaction and emotional disengagement among university students can foster psychosocial challenges such as loneliness (Tomé et al., 2016). The unpredictability surrounding career prospects may intensify these difficulties and negatively influence academic outcomes and general well-being.

Career concerns involve managing uncertainties and apprehensions related to one’s professional future. These concerns are significantly associated with individuals’ emotional adjustment, including increased experiences of loneliness and academic alienation (Amarat et al., 2019; Bell et al., 1990). In particular, career-related anxiety is believed to influence not only students’ psychosocial state but also the strength of the associations between career goal inconsistency and adverse emotional outcomes. How career concerns shape students’ social connections and emotional resilience is a domain that warrants further investigation (Rokach, 2019).

This study seeks to thoroughly investigate the interrelationships among career goal inconsistency, career concerns, loneliness, and university alienation. The research was conducted among university students in sports sciences programs, who may be particularly vulnerable to uncertainty and instability in their professional trajectories. These conditions may elevate career-related anxiety, which in turn can affect students’ emotional experiences and academic engagement.

The findings of this research are expected to contribute to the literature by addressing theoretical gaps and providing practical insights for improving student support in career planning. Furthermore, the results may assist educational institutions in designing more effective interventions to promote students’ psychosocial well-being and academic success.

### The association with career goal inconsistency with loneliness and alienation

Career goal inconsistency is the discordance between an individual’s professional aspirations and current circumstances. Meta-analytic evidence indicates that, in general, goal conflict is significantly associated with poorer psychological well-being, including increased psychological distress and lower life satisfaction (Creed et al., 2015; Gray et al., 2017).

The evidence indicates that inconsistency in job goals correlates with the deterioration of social connections and heightened feelings of loneliness (Jawahar and Shabeer, 2019). This inconsistency can hinder students’ adjustment processes by diminishing their perceived control over career decisions (Marini et al., 2023; Hirschi and Vondracek, 2009). Inconsistency in career objectives may be associated with heightened feelings of isolation by prompting individuals to distance themselves from their social surroundings. Difficulties in career planning procedures induce isolation, which intensifies when individuals lack adequate social support mechanisms (Creed et al., 2015; Coetzee and Schreuder, 2018). Inconsistency in career goals is linked to alienation from the university. University alienation is when an individual experiences disconnection from academic and social environments, resulting in a diminished sense of belonging. Inconsistency in professional objectives may be associated with feelings of exclusion or isolation within the educational setting. This sentiment may intensify in contexts characterised by economic and social disadvantages (Marini et al., 2023).

This phenomenon is particularly relevant in academic disciplines with uncertain employment pathways, where students from socially or economically disadvantaged backgrounds may experience amplified career anxiety and detachment from the educational environment (Muzika et al., 2019).

Despite growing evidence linking career goal inconsistency to various psychosocial risks, empirical studies have rarely examined its differentiated impact on loneliness and academic alienation within a single framework. Most existing research treats these constructs in isolation, overlooking their potential co-occurrence and shared antecedents—especially in populations experiencing structural career uncertainty, such as sports sciences students. By addressing this gap, the current study aims to reveal whether perceived discrepancies in career direction are simultaneously predictive of students’ social–emotional disconnection and institutional detachment.

*H1*: Inconsistency in career goals will be positively associated with loneliness.

*H2*: Inconsistency in career goals will be positively associated with alienation from the university.

### The influence of career considerations: direct and indirect impacts

Career concerns significantly influence the psychosocial experiences of university students, particularly at this pivotal phase of their professional development. These concerns frequently manifest as anxiety around future employment opportunities and may result in adverse emotions such as loneliness and alienation. Research indicates that both state and trait anxiety are significantly correlated with career hesitation among students, impairing their capacity to make confident professional choices (Mojgan et al., 2011). Career indecision may exacerbate feelings of loneliness by prompting students to withdraw from social contacts owing to apprehensions about the future. In this context, anxiety and loneliness create a reciprocal loop that reinforces one another. (Ghiggia et al., 2023; Keller et al., 2022).

The connection between career apprehensions and loneliness can be more effectively comprehended through emotional competencies and social attachment mechanisms. Individuals suffering from career anxiety may struggle with emotional management abilities, hindering their ability to form stable relationships and engage in social settings (O’Day et al., 2019). This lack of participation can result in a sense of alienation, causing individuals to feel separated from their classmates and the academic community. A sense of belonging is crucial during university years, and when job aspirations are ambiguous, students may experience heightened isolation (Wright et al., 2014; Potgieter et al., 2021). Loneliness in this setting may significantly mediate students’ emotional suffering concerning job worries. Students suffering from professional concerns may exacerbate their loneliness and alienation through social retreats (Moeller and Seehuus, 2019).

The disparity between individuals’ job aspirations and present circumstances might be associated with heightened career anxieties and diminished self-confidence in attaining professional success (Miller and Rottinghaus, 2013). Recent European evidence also suggests that perceived discrepancies between career expectations and real opportunities can reduce self-efficacy and confidence in achieving career success (Gómez-Jorge and Díaz-Garrido, 2023). This discrepancy may result in ambiguity concerning professional trajectories and foster emotions of inadequacy and isolation in individuals. Studies indicate that persons who identify more significant work obstacles often experience more loneliness due to a sense of lack of support (O’Day et al., 2019; Keller and Brown, 2013). The significance of social support is paramount; individuals who pursue support in their professional endeavours experience elevated work well-being and diminished feelings of loneliness (Wright et al., 2014; Cutrona and Russell, 1990). Moreover, recent empirical evidence highlights a robust positive relationship between perceived social support and academic engagement, mediated via enhanced life satisfaction and academic motivation (Chen et al., 2023).

Although prior studies have investigated career anxiety, its dual impact on both loneliness and institutional alienation remains under-theorised. Furthermore, how career goal inconsistency operates as an antecedent of such worries has not been systematically explored in student populations experiencing structural professional uncertainty. The present study aims to illuminate how professional concerns shape emotional and academic detachment by addressing this limitation.

This study investigates the impact of professional worries on loneliness and university alienation, as well as how inconsistencies in career goals predict these concerns, by the following hypotheses:

*H3*: Career concerns will be positively associated with loneliness.

*H4*: Career concerns will be positively associated with university alienation.

*H5*: Career goal inconsistency will be positively associated with career concerns.

### The mediating role of career concerns

Inconsistency in career goals correlates with psychosocial effects, including loneliness and academic alienation, by heightening individuals’ uncertainties regarding the future. Nevertheless, job considerations emerge as a significant factor influencing these interactions. Career issues may serve as a mechanism that strengthens the association between individuals’ anxiety regarding their professional future and feelings of loneliness and alienation (Hu et al., 2017; Jawahar and Shabeer, 2019).

Career-related apprehensions may diminish individuals’ social connections by amplifying their emotional strain. Career apprehensions may be associated with individuals’ retreat from social engagements, exacerbating feelings of isolation. The study by Hu et al. (2017) demonstrates that career-related stress is associated with individuals abandoning their goals, leading to withdrawal behavior linked to feelings of loneliness. Consequently, the effects of career-related issues on personal and social life influence a person’s perception of loneliness. We anticipate that career considerations may mediate the incompatibility of career objectives associated with university alienation. According to a study by Creed et al. (2015), differences in professional goals are associated with lower commitment to the academic community, which is linked to higher anxiety and a weaker sense of belonging. Individuals experiencing elevated anxiety levels may withdraw from the collegiate setting, resulting in a heightened sense of alienation.

Based on the following hypotheses, which were structured to ensure conceptual clarity and methodological rigour and drew on the hypothesis structuring approach and methodological clarity employed by Rehman et al. (2024), this study investigates how professional anxiety might play a part in the links between loneliness, feeling alone at university, and having conflicting career goals.

*H6*: Career concerns will mediate the association between career goal inconsistency and loneliness.

*H7*: Career concerns mediate the association between career goal inconsistency and university alienation.

This study investigates the relationships among career goal inconsistency, career concerns, loneliness, and university alienation, explicitly focusing on the mediating role of career-related anxieties. While these constructs have been individually studied in various contexts, there remains a significant gap in understanding how they interact within the domain of sports sciences education, especially under conditions of occupational uncertainty.

In this regard, students enrolled in faculties of sports sciences in Turkey represent a particularly relevant population. According to the Council of Higher Education (YÖK, 2024), approximately 56,000 students are currently enrolled in sports sciences faculties and an additional 18,000 in physical education and sports colleges. Despite their potential to meet the country’s workforce needs in the sports sector, these students often face substantial employment uncertainty after graduation.

The Public Personnel Selection Examination (KPSS), a critical structural factor shaping this uncertainty, is a national requirement for public school teaching positions. In 2023, only 320 teaching positions were allocated for 26,346 candidates applying in physical education (MEB, 2024), reflecting a highly competitive and unstable career landscape. This competitive environment amplifies students’ concerns about future employability, which may contribute to feelings of loneliness and alienation from academic life.

Given these dynamics, Turkey presents a salient case for exploring how structural career uncertainties intersect with students’ psychosocial experiences. The present study aims to contribute theoretically and practically by identifying the psychological mechanisms through which career goal inconsistency affects emotional and academic outcomes, offering implications for institutions facing similar challenges worldwide.

## Method

### Participants and procedure

GPower 3.0.1. programme was used to determine the participants. As a result of the multiple regression-based power analysis for mediation analysis in the context of Structural Equation Modelling (SEM), it was determined that at least 129 participants were required according to the criteria *α* = 0.05, medium effect size (*f*^2^) = 0.15 and power (1−*β*) = 0.95. Pieters (2017) stated that the minimum sample size should be at least 450 to detect indirect effects using a bias-corrected confidence interval. This study’s participants consisted of 631 students, 352 males (55.8%) and 279 females (44.2%), studying in the faculties of sports sciences. The average age of the students in the participants was (*n* = 631, 21.75 ± 4.601). On the other hand, 133 (21.1%), 190 (30.1%), 146 (23.1%), and 162 (25.7%) of the students were studying at the 1st-grade, 2nd-grade, 3rd-grade, and 4th-grade levels, respectively.

### Data collection

Data were collected via an online survey form created using Google Forms. The survey link was distributed to students enrolled in the Faculty of Sport Sciences through official university mailing lists and faculty social media groups. Participation was voluntary, and all participants provided informed consent before the survey began. No personally identifying information was collected, and responses were kept strictly confidential. The data collection period lasted from 20 December 2024 to 1 February 2025. Upon completion of the survey, the anonymised dataset was stored in a secure, password-protected repository accessible only to the research team for analysis purposes.

### Instruments

To assess the potential impact of standard method bias, we conducted Harman’s single-factor test by entering all items into an unrotated exploratory factor analysis. The first factor accounted for 30% of the total variance, well below the 50% threshold, suggesting that common method bias was unlikely to be a significant issue (Podsakoff et al., 2003). Moreover, procedural remedies, such as guaranteeing anonymity, randomising item order, and varying scale formats, were applied during the survey design to mitigate standard method variance further.

### The university alienation index

The instrument, the validity and reliability of which was carried out by Kurtulmuş et al. (2015) to measure the alienation levels of students to university, has a one-dimensional structure and consists of 9 items. The instrument was designed as a 5-point Likert-type. The instrument has items such as ‘*I do not think I am a part of this university*’ for measuring students’ alienation from the university. On the other hand, it was stated that no item was included in reverse scoring in the instruments. CFA was conducted to test the validity of the university alienation scale within the scope of the current study. As a result of the analysis, covariance was performed between questions 3 and 4, and the results showed acceptable fit values x2 = 86.31/Df = 26, CFI = 0.98, TLI = 0.97, NFI = 0.97, IFI = 0.98, GFI = 0.99, RMSEA = 0.061, SRMR = 0.028, Coefficient *ω* = 0.90, Coefficient *α* = 0.89. The factor loadings of the items in the University Alienation Index ranged between 0.51 and 0.88.

### UCLA loneliness scale

The measurement instrument, modified in abbreviated form for Turkish culture by Doğan et al. (2011), comprises eight components and exhibits a unidimensional structure. The measurement instrument employs a 4-point Likert scale, with two items scored in reverse. The measurement instrument comprises inquiries such as ‘I have no friends’ and ‘I feel alone from other people’ to assess individuals’ levels of loneliness. Confirmatory Factor Analysis (CFA) was performed to evaluate the construct validity of the loneliness scale in the present study. As a result of the analysis, covariance was performed between questions 1 and 2, 3 and 4, and it was determined that the results showed acceptable fit values x2 = 64.991/Df = 18, CFI = 0.966, TLI = 0.95, NFI = 0.95, IFI = 0.97, GFI = 0.99, RMSEA = 0.06, SRMR = 0.03, Coefficient *ω* = 0.89, Coefficient *α* = 0.90. Standardised factor loadings for the items of the Loneliness Scale ranged from 0.40 to 0.68, indicating acceptable item–construct relationships.

### Career goal inconsistency

The scale created by Creed and Hood (2015) was translated into Turkish by Yam et al. (2020) to assess university students’ career goal inconsistency levels. The Turkish adapted scale comprises 12 items and possesses a unidimensional structure. The instrument does not include any items for reverse scoring. The measurement instrument was constructed as a 7-point Likert scale. To assess the degrees of career goal inconsistency among university students, the instrument incorporates questions such as, ‘The accomplishments I have attained thus far do not instil confidence in my ability to fulfil my professional objectives.’ In the context of the present study, we evaluated the construct validity of the career aim inconsistency scale using Confirmatory Factor Analysis (CFA). As a result of the analysis, covariance was performed between questions 2 and 3, 4 and 5, 10 and 11, and the results showed acceptable fit values x2 = 288.14/Df = 52, CFI = 0.95, TLI = 0.94, NFI = 0.944, IFI = 0.95, GFI = 0.97, RMSEA = 0.08, SRMR = 0.04, Coefficient *ω* = 0.91, Coefficient *α* = 0.92. Standardised factor loadings for the Career Goal Inconsistency Scale items ranged from 0.56 to 0.88, indicating substantial and statistically significant item–construct relationships.

### Adult career concerns scale

The 12-item short form of the measurement test created by Perrone-McGovern et al. (2003) was translated into Turkish by Şahin et al. (2019) to assess the professional concerns of adult persons. The Turkish-adapted scale comprises a total of 9 elements. The measurement instrument comprises four sub-dimensions: exploration, maintenance, establishment, and withdrawal. The measurement instrument was constructed using a 5-point Likert scale, and it was indicated that no items required reverse scoring. The data-collecting instrument, designed to assess the degree of career-related apprehensions among adults, includes topics such as *‘Identifying a profession that captivates me’ and ‘Strategising my advancement within my current industry*.’ This study employed the adult career concern scale in a unidimensional framework. The construct validity of the assessment instrument in this study was evaluated using confirmatory factor analysis (CFA). A secondary-level confirmatory factor analysis (CFA) was done to show that the scale items were valid in a one-dimensional framework. This was done to check the construct validity of the instrument. As a result of the analysis, it was determined that the results showed acceptable fit values x2 = 94.96/Df = 23, CFI = 0.98, TLI = 0.97, NFI = 0.98, IFI = 0.98, GFI = 0.99, RMSEA = 0.07, SRMR = 0.03, Coefficient *ω* = 0.95, Coefficient *α* = 0.92. Standardised factor loadings for the Adult Career Concerns Scale items ranged from 0.64 to 0.81, confirming acceptable item–construct relationships.

### Data analysis

Initially, the data were imported into the JASP 0.16.4 statistical software. Subsequently, the dependability of the measurement model was evaluated. The values of Cronbach’s Alpha, McDonald’s Omega, *χ*^2^/df, CFI, TLI, NFI, GFI, SRMR, and RMSEA were examined. After evaluating the measurement model, the mean, standard deviation, skewness, and kurtosis values were analysed. Values between ±3 for skewness and kurtosis were used as standards for the normality test (Kline, 2016). We performed a Pearson correlation analysis to determine the relationships between loneliness, alienation from university, job concerns, and career goal inconsistency. We used the SEM mediation part of the JASP program to test the model based on the hypotheses. This SEM-based analytical strategy aligns with previous educational and psychosocial studies employing structural equation modelling to explore direct and mediated effects (Rehman et al., 2025; Uğraş et al., 2025). The study used bootstrap analysis to determine the direct and indirect associations related to the model’s variables (Preacher and Hayes, 2008). In the bootstrap analysis, 5,000 samples were utilised. The model’s variables were judged to have significant direct and indirect effects when they were not zero at the lower and upper ends of the 95% confidence interval (Preacher and Hayes, 2008).

## Ethical approval

The ethics committee of the study, numbered 2024-SBB-0911, was obtained from Bartın University’s Social and Human Ethics Committee. The sample group was informed before collecting the data and acquiring consent forms.

## Results

Table 1 presents the variables’ correlations, mean, standard deviation (SD), skewness and kurtosis values. The mean scores and standard deviations of the instruments were determined as follows: CGD (M = 3.13, SD = 1.31), CC (M = 2.53, SD = 1.05), L (M = 1.82, SD = 0.53), AU (M = 2.31, SD = 0.99). On the other hand, kurtosis and skewness values of the Instruments were found as CGD 0.42 and −0.29, CC 0.29 and −0.63, L 0.99 and 0.43, AU 0.64 and −0.27. When the relationships between career goal inconsistency (CGD), career concerns (CC), loneliness (L) and alienation from university (AU) were examined, it was found that there was a significant positive correlation between CGD and CC (*r* = 0.060, *p* < 0.001), and a significant positive correlation between L and AU (*r* = 0. 34, *p* < 0.001) positively significant, CC and L positively significant (*r* = 0.34 *p* < 0.001), CGD and L positively significant (*r* = 0.38 *p* < 0.001), CGD and AU (*r* = 0.43 *p* < 0.001) (see Table 2).

**Table 1:** Kurtosis, skewness, mean scores, standard deviation scores and correlational relationships of instruments.

Variables	CGD	CC	L	AU	M	SD	Skewness	Kurtosis
CGD	–			–	3.13	1.31	0.42	−0.29
CC	0.60**	–		–	2.53	1.05	0.29	−0.63
L	0.38**	0.34**	–	–	1.82	0.53	0.99	0.43
AU	0.43**	0.35**	0.34**	–	2.31	0.99	0.64	−0.27

**Indicates that the correlation is statistically significant at the *p* < 0.01 level.

**Table 2:** Direct, indirect and total association results.

Association type	Predictor → outcome	Estimate	Std. error	*z*-value	*p*-value	95% CI lower	95% CI upper
Direct association	CGD → L	0.21	0.03	6.17	<0.001	0.15	0.28
	CGD → AU	0.26	0.03	7.64	<0.001	0.19	0.33
	CC → L	0.17	0.05	3.71	<0.001	0.08	0.26
	CC → AU	0.15	0.04	3.29	<0.001	0.06	0.23
	CGD → CC	0.46	0.02	18.86	<0.001	0.41	0.50
Indirect association	CGD → CC → L	0.08	0.02	3.64	<0.001	0.04	0.12
	CGD → CC → AU	0.07	0.02	3.25	0.001	0.03	0.11
Total association	CGD → L	0.29	0.03	10.39	<0.001	0.24	0.35
	CGD → AU	0.33	0.03	11.92	<0.001	0.27	0.38

**Figure 1:**
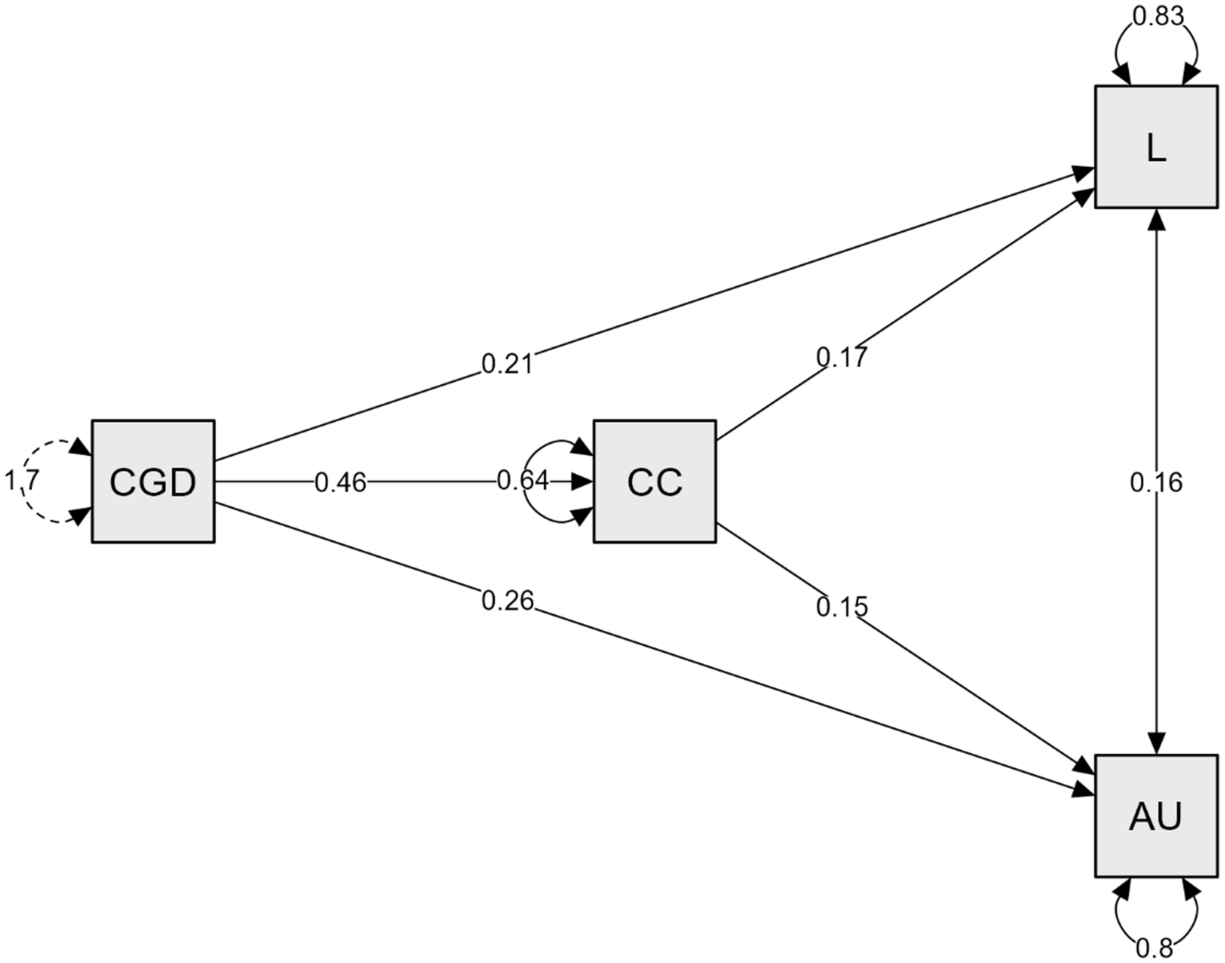
Path analysis (CGD, Career Goal Inconsistency; CC, Career Concerns; L, Loneliness; AU, Alienation from University). Arrows represent associative paths in cross-sectional SEM; they do not imply causality.

The direct association with career goal inconsistency on loneliness [*B* = 0.21, *z* = 6.17, *p* < 0.001, CI(0.15, 0.28)] is significant, the direct linked to on university alienation (*B* = 0.26, *z* = 7.64, *p* < 0.001, CI[0.19, 0.33]) is significant and the direct related to on career concerns (*B* = 0.46, *z* = 18.86, *p* < 0.001, CI[0.41, 0.50]). The mediating association with career concerns on the relationship between career goal inconsistency and loneliness [*B* = 0.08, *z* = 3.64, *p* < 0.001, CI(0.04, 0.12)] was found to be significant. On the other hand, the mediating association with career concerns on the relationship between career goal inconsistency and university alienation [*B* = 0.07, *z* = 3.25, *p* = 0.001, CI(0.03, 0.11)] was significant. The total association with career goal inconsistency on loneliness [*B* = 0.29, *z* = 10.39, *p* < 0.001, CI(0.24, 0.35)] was significant, and the total association with career goal inconsistency on university alienation [*B* = 0.33, *z* = 11.92, *p* < 0.001, CI(0.27, 0.38)] was significant. The variance percentages explained for the dependent variables in the research model are as follows: Loneliness (L): *R^2^* = 0.16 (16% explained variance), Alienation from university (AU): *R^2^* = 0.20 (20% explained variance), Career concerns (CC): *R^2^* = 0.36 (36.0% explained variance) (see Figure 1).

## Discussion

This study investigated the correlations between career goal inconsistency, loneliness, alienation, and career concerns. The results indicated that inconsistencies in career goals are positively associated with loneliness and alienation and that career concerns statistically account for part of this association. The study facilitates understanding of how cognitive inconsistencies related to career intertwine with psychosocial outcomes during the transition to adulthood. The discussion interprets the findings within the existing literature framework while highlighting the theoretical and practical implications of these complex relationships.

One of the study’s key findings is that inconsistencies in career goals are significantly associated with loneliness and alienation from university. The conflicts individuals experience between their internal orientations and external expectations, such as family, societal, or systemic pressures, can be associated with emotional distress and weakened social bonds. Indeed, in Widyowati et al. (2024) study, the mismatch between parental expectations and individual career aspirations was identified as a factor increasing loneliness and stress levels. Creed and Hood (2015) observed that inconsistencies in career goals can result in social withdrawal and attachment issues. These findings show that loneliness is related to the individual’s withdrawal from their social environment and internal conflict and decision uncertainty. Haase et al. (2012) study emphasises the relationship between emotional states and career motivation. It is stated that positive emotions increase goal orientation, while negative emotions reduce effort and deepen feelings of loneliness. On the other hand, these inconsistencies in career goals weaken not only the individual’s emotional integrity but also their sense of belonging to the academic environment. Our research findings show that individuals who experience inconsistencies in their career goals have higher levels of alienation from the university environment. Botha and Mostert’s (2013) study argues that inconsistencies in career goals, combined with environmental stress factors, trigger burnout and alienation in students. Ketonen et al. (2016) note that individuals with low self-regulation skills experience weakened academic belonging. The theoretical framework presented by Hascher and Hadjar (2018) reveals that a lack of academic attachment can result in failure and alienation from the education system. Our findings are consistent with this theoretical framework. Internal inconsistencies individuals experience regarding their career goals weaken their commitment to the current educational environment and prevent them from perceiving the university as an educational institution and a place of belonging.

The findings of this study reveal that career concerns have a significant and positive association with both loneliness and alienation levels among university students. Students’ feelings of insecurity about the future and career concerns not only increase their internal anxiety levels but also weaken their social relationships and disrupt their sense of belonging to the academic environment. Nuckols et al. (2023) study indicates that loneliness is directly related to individuals’ financial and career concerns; this relationship is further strengthened by a lack of social support and a perception of insecurity. Similarly, Yousefi et al. (2011) found that anxieties and decision-making difficulties experienced during career planning can disrupt individuals’ psychological adjustment and increase feelings of loneliness. The findings of Zheng et al. (2021) also show that individuals’ social bonds weaken and their levels of loneliness increase, especially in conditions where career-related stress increases. However, career concerns are directly associated with the individual’s emotional state, perception of the university environment, and sense of belonging. Our research findings show that individuals with high levels of career anxiety experience a significant increase in their level of alienation from the university. Students may lose interest in and commitment to the academic environment due to their career-related concerns, beginning to view the university merely as a stepping stone or a mandatory obligation. This situation leads to a decrease in the university’s affiliation and deepening feelings of alienation. Findings in the literature also support this relationship. Tien et al. (2005) study stated that career-related concerns weaken students’ emotional attachment to the university, particularly reducing their motivation for the academic process. Kwok (2018) argues that job insecurity, uncertain career expectations, and economic concerns weaken students’ connections to the university environment and increase alienation. Similarly, Talib and Aun (2009) stated that insecurity in the career decision-making process is negatively linked to individuals’ academic motivation and organisational commitment. In the context of Turkey, this relationship becomes even more pronounced. In particular, the competitive pressure brought about by centralised exams, such as the Public Personnel Selection Exam (KPSS), limited public employment, and structural insecurity create intense career anxiety among students, causing them to become alienated from the educational process and weaken their sense of belonging to the university. Therefore, career anxiety is not merely an individual psychological state; it can also be considered a multi-layered risk factor that links emotional attachment to and participation in university life. At this point, it is understood that career anxiety is not only linked to loneliness and alienation but also that career goal inconsistency plays a decisive role in its formation. Our findings show that inconsistencies in career goals are associated with higher levels of career anxiety in individuals. Kwok’s (2018) study states that a lack of uncertainty management leads to inconsistencies in individuals’ career goals, increasing professional anxiety. Similarly, Xu and Bhang’s (2019) research reveals that individuals with unclear career goals encounter more difficulties in decision-making, a lack of information, and interpersonal conflicts, which increase psychological pressure and raise anxiety levels. In this context, career anxiety can be seen not only as a result but also as a multifaceted process fuelled by uncertainty about career goals and association with the individual’s psychosocial adaptation.

Our research findings reveal that career concerns mediate the association between career goal inconsistency and loneliness and alienation from university. Inconsistency in career goals weakens individuals’ confidence in their future and creates grave uncertainty about their professional development. This uncertainty increases career anxiety in individuals and ultimately is associated with psychosocial outcomes such as loneliness and alienation. Hu et al. (2016) emphasise that the mismatch between career goals and performance perceptions is associated with increased stress and career-focused anxiety and may contribute to indirect psychological consequences. It has been stated that negative feedback regarding goals can increase individuals’ concerns about their careers, weaken their social bonds, and reinforce feelings of loneliness. Similarly, Widyowati et al. (2024) have pointed out that inconsistencies between individuals’ career goals and parental expectations may be associated with loneliness and social isolation. The theoretical framework presented by Xu and Tracey (2014) also reveals that individuals with low tolerance for uncertainty experience greater career anxiety in their career decision-making processes. This situation can negatively affect individuals’ psychological adjustment. These studies reveal that career goal inconsistencies do not directly associate with loneliness but rather primarily through career anxiety. Our findings are consistent with this indirect association with the mechanism and point to a direct association. Similarly, career goal inconsistency indirectly associates with alienation from the university by increasing career anxiety. The discrepancies between students’ career goals and their current academic context increase their uncertainty about the future, which triggers career concerns. Increased career anxiety weakens individuals’ sense of belonging to the university and leads to alienation from the academic process. Kwok (2018) states that individuals who are inadequate in managing uncertainty experience greater career goal inconsistency, which is negatively associated with their educational and social commitment levels and increases alienation. Hirschi’s (2009) study also shows that individuals with low career adjustment ability feel less control over the career planning process, which can weaken academic attachment and increase feelings of alienation. Bullock-Yowell et al. (2012) study reveals that negative career thoughts weaken individuals’ decision-making self-efficacy, which, together with growing career concerns, undermines commitment to academic processes and intensifies feelings of alienation. All these findings suggest that career goal inconsistencies are associated with alienation from university directly and indirectly through career concerns.

## Limitations of the study and suggestions for further studies

This study’s findings yield recommendations for future research and applications about the interrelations among career goal inconsistency, loneliness, university alienation, and career concerns. Exploring the manifestations of job goal inconsistency and related issues of loneliness and alienation across various cultural and social situations is necessary. In addition, longitudinal studies should be conducted to assess the temporal changes in these associations over time, enabling a more dynamic understanding of the observed relationships.

Another limitation of this study is its cross-sectional design. As all variables were measured concurrently, it was not possible to establish temporal order, which limits causal inference. Although Harman’s single-factor test indicated that common method bias was not a significant issue, future research should use longitudinal or experimental designs to clarify causal relationships. Therefore, the mediation model tested in this study should be interpreted as conceptual and exploratory, reflecting associations consistent with the proposed theoretical framework rather than definitive evidence of causal pathways.

While mediation analysis with cross-sectional data has inherent limitations, it may still provide valuable insights under certain conditions, such as when resources are limited or when examining emerging constructs (Disabato, 2016; Jose, 2017; Hayes, 2022). A further limitation concerns the exclusive use of self-report questionnaires administered online. This method may introduce social desirability bias and standard method variance, potentially inflating the observed associations. Future studies could reduce such risks by adopting multi-method approaches or collecting data at different times.

The generalisability of the findings is also limited, as the sample consisted solely of students from the faculty of sports sciences. Results may not fully apply to students from other disciplines or the general population. Finally, the study did not examine other psychosocial or environmental factors influencing career goal inconsistency, loneliness, and alienation.

Moreover, it is essential to consider the specific sociocultural and institutional context of Türkiye when interpreting these findings. In particular, the high-stakes national Public Personnel Selection Examination (KPSS), combined with limited government employment quotas, may substantially contribute to students’ heightened levels of career anxiety and academic alienation. These institutional realities are not necessarily transferable to other countries or contexts, and future studies should be mindful of such systemic and structural differences when generalising findings beyond the Turkish higher education setting.

## Conclusion

In conclusion, our findings suggest that the association between career goal inconsistency and university alienation is better understood through psychological considerations, including career anxieties. Elevated anxiety levels diminish individuals’ engagement in academic activities, hence impairing their sense of belonging within the university setting.

This study investigates the interconnections among career goal inconsistency, loneliness, university alienation, and professional worries within a multidimensional framework. The results show that having inconsistent career goals is associated with higher levels of loneliness and alienation in school and is associated with reduced social participation and weaker academic belonging. Inconsistencies between career aspirations and perceived realities are associated with reduced social participation and weaker academic belonging. This study substantially contributes to comprehending these links from theoretical and practical viewpoints.

Beyond theoretical insights, the findings also offer meaningful practical implications. University career counsellors should develop early detection systems to identify students showing signs of career goal inconsistency and anxiety. Incorporating structured guidance interventions, such as personalised career planning sessions and psychosocial support workshops, may reduce students’ feelings of loneliness and academic alienation. Furthermore, institutions may benefit from integrating peer mentoring and faculty advising systems to foster a stronger sense of belonging, particularly in academic programs with less stable professional pathways, such as sports sciences.

## Data Availability

The datasets presented in this study can be found in online repositories. This data can be found here: bit.ly/3K9miPM.
